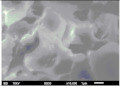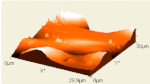# Properties and Characterization of New Approach Organic Nanoparticle-Based Biocomposite Board

**DOI:** 10.3390/polym12102236

**Published:** 2020-09-28

**Authors:** I. Ismail, Arliyani, Z. Jalil, Mursal, N. G. Olaiya, C. K. Abdullah, M. R. N. Fazita, H. P. S. Abdul Khalil

**Affiliations:** 1 Physics Department, Mathematics and Natural Sciences Faculty, Universitas Syiah Kuala, Banda Aceh 23111, Indonesia; arlianikhatijah@gmail.com (A.); zjalil@unsyiah.ac.id (Z.J.); mursal@unsyiah.ac.id (M.); 2 School of Industrial Technology, Universiti Sains Malaysia, 11800 Penang, Malaysia; ngolaiya@futa.edu.ng (N.G.O.); fazita@usm.my (M.R.N.F.)

**Keywords:** composite, coconut shell, resin epoxy, ball-milling, nanoparticle

## Abstract

Conventionally, panel boards are produced with material flex or microparticle with P.U. or U.F. as adhesives. However, in this study, nanoparticle with epoxy resin as an adhesive was used to produce nanoboard. Coconut shell nanoparticle composite with epoxy resin as an adhesive was prepared using a compression molding technique. The coconut shell particles were originally 200 mesh size and then milled mechanically with a ball mill for the duration of 10, 20, 30, and 40 h (milling times) to produce nanoparticles. The composition ratio of the composite is 85 vol.% of coconut shell and 15 vol.% of epoxy resin. The formation of nanoparticles was observed with transmission electron microscopy (TEM). The mechanical, physical, and microstructure properties of the composite were examined with X-ray diffraction, scanning electron microscopy, atomic force microscopy, and universal testing machine. The results established that the properties of the composite (microstructures, mechanical, and physical) are influenced by the duration of milling of coconut shell particles. The modulus and flexural strength of the composite improved with an increase in the milling time. The density, thickness swelling, and porosity of the composite were also influenced by the milling times. The result suggested that the composite properties were influenced by the particle size of the coconut shell. The coconut shell nanoparticle composite can be used in the manufacturing of hybrid panels and board.

## 1. Introduction

The use of polymer composites for industrial applications has been on the increase. Polymer composites have been used for several applications, such as aircraft, automotive, household appliances, electronic devices, etc. The use of flex and microparticle with phenol-formaldehyde and urethane formaldehyde as adhesive to produce panel board has been established. Previous work showed that these boards could not be used for applications that require high strength. The properties of these boards are quite low. Epoxy resin has been used for high strength matrix and high-performance composite. The new approach established in this study is the use of nanoparticle with epoxy as an adhesive to produce high strength nano board.

Traditionally, composites are made from fossil fuels, and due to the environmental pollution resulting from long time degradation process, scientists have developed composites from renewable materials [1,2]. One of the solutions that have been proposed to solve the challenge of degradation of synthetic polymers is to blend them with renewably sourced materials [3]. A blend of synthetic and renewable polymers to enhance the degradation properties of synthetic polymers are either binary or ternary blend composites and has attracted a lot of interest [4,5,6,7,8]. Sustainable reinforcements or fillers for biocomposite are from natural fibers (such as bamboo, wood, cotton, ramie), agro and forestry residues (such as rice straw, wheat straw, coconut shell), industrial co-products (such as bagasse, coir, husk), and recycled fibers (such as cardboard and carpet) [4]. Biocomposites are very promising to overcome environmental problems [9]. However, obtaining good mechanical properties is very challenging because of the compatibility between hydrophobic synthetic polymers and hydrophilic biomass [4,5,6].

Coconut is a widely grown plant in Indonesia, Philippines, India, Sri Lanka, Thailand, Malaysia, and other tropical countries. In 2018, there were about 61 million tons of coconuts produced worldwide [10]. Indonesia is the largest country providing coconut in the world. In 2018, Indonesia, Philippines, and India produced 19, 14, and 12 million tons of coconuts, respectively [10]. One of the by-products (residues) of coconut production is coconut shell. Thus, there is quite a lot of coconut shell waste in Indonesia, the Philippines, and India that can be used as a filler for biocomposite. Coconut shells contain 34% of cellulose, 21% of hemicellulose, and 27% of lignin [11]. Bledzki et al. produced a composite with a coconut shell microparticle and polypropylene as a matrix. Their report concluded that the coconut shell could be used for composite [11]. Leman et al. proposed that concrete can be filled by a coconut shell [12]. Chun et al. studied the properties of biocomposite from coconut shell particles using a polylactic acid matrix. It was reported in their work that the biocomposite of coconut shell particle has good mechanical properties, which were significantly influenced by the addition of the nanoparticles [13]. Vasu et al. studied the composite made of high-density polyethylene filled by coconut shell particles. The composite was reported to have excellent mechanical properties because of the coconut shell filler [14]. Several studies on coconut shell powder composite with epoxy resin have been conducted. Singh et al. used micro size coconut shell particles to form a composite with the epoxy. Their report showed that the microparticles enhancement improved the flexural strength and density of the composite [15]. Somashekhar et al. combined coconut shell powder and tamarind shell powder to form a composite by using the epoxy matrix. It was found that the tamarin shell powder improved the mechanical properties of the composite [16]. Durowaye et al. also combined coconut shell powder (40 mesh) with palm fiber to form a composite by using the epoxy resin matrix. However, the composite flexural strength reported composite was rather low when compared with similar studies [17]. Chary et al. [18] fabricated the composite using coconut shell particles with the epoxy resin and melamine matrix. The performance of the mechanical properties of their biocomposite improved as the particle size of the coconut shells was reduced from 10 mesh to 60 mesh [18]. However, most studies focused on the use of micro-sized coconut shells. Furthermore, no detailed studies of microstructure, mechanical, and physical properties of coconut shell nanoparticles with epoxy resin have been reported.

At present, studies on particle size analysis have been developed for the use of nanoparticles. It has been reported that nanoparticles are strong reinforcement in polymers for improved mechanical properties. Nanocomposite provides excellent performance, which offers an exceptionally extensive range of prospective applications [19,20]. Therefore, it is very interesting to study biocomposites for particle size in nanoscale. Nanoparticle production with a mechanical planetary ball mill has been proposed and used [21,22,23]. Several parameters are required for the optimum production of nanoparticles using a planetary ball mill [24,25]. One of them is milling time. Hewitt et al. showed that the physical properties of the composite are affected by the duration of milling [22]. Singh et al. also reported that the milling time of the machine significantly affects the properties of Ti4Al alloy [26]. The previous study on the milling time of coconut shell particles showed that there is a significant effect on the chemical properties of coconut shell particles [27]. The change in the chemical properties of the coconut shell was due to the change in the percentage composition of Fe_2_O_3_ in coconut shell particles with the milling time [27]. The change in composition and particle size changed the mechanism of coconut filler-matrix bonding, and this formed a new composite with different properties and characterization from those previously reported. The change is due to either chemical reactions, mechanical interaction, or physical interaction between coconut filler-matrix.

Several studies have been conducted on coconut shells as fillers in epoxy polymer composite (high matrix), but there are no studies on coconut shell nanoparticle biocomposite with epoxy as a binder (low matrix). Established studies have been done using the composition of epoxy with a higher percentage as the matrix and natural fiber as a filler (smaller percentage) to produce a high-performance composite (high matrix with low filler), i.e., sheet-molding compound (S.M.C.), bulk molding compound (B.M.C.), etc. The neat epoxy resin is brittle and has low-impact toughness, but with the addition of a small amount of natural filler, the epoxy resin becomes strong and has very good fatigue strength.

In this study, low matrix with high filler, known as the conventional composite (or biocomposite), which is low-performance composite, is prepared. In order to achieve good performance for biocomposite, the filler particle size is changed to nano size, which improved the surface area with the low composition of the matrix (epoxy resin) to produce biocomposite.

## 2. Materials and Methods

### 2.1. Materials

The dried coconut shell was milled by PT Indratma Sahitaguna, Indonesia, to produce coconut shell particles (200 mesh of powder). The epoxy resin obtained from Hexion Specialty Chemicals Sdn. Bhd, Penang, Malaysia was used for matrix.

### 2.2. Preparation of Coconut Shell Nanoparticles

The coconut shell (200 mesh of powder) was dried at 80 °C. A planetary ball mill produced by Fritsch Germany was used to mill the coconut shell particles to produce nanoparticles of coconut shells. The ball to powder ratio was 2:1 (wt.). The speed of milling was 350 rpm (constant). The duration of milling was varied from 10, 20, 30, to 40 h with the initial size as the control at 0 h.

### 2.3. Preparation of Composites

The coconut shell nanoparticle board was produced with a compression molding technique. Coconut shell particles (C.S.P.) that had been ground with the ball mill into nano sizes were mixed with epoxy resin for 30 min with a Rheomixer at a constant speed (300 rpm). The composition variation of the mixture was 85% by volume of the coconut shell nanoparticles and 15% epoxy resin which was determined based on the previous literature [28]. The blend was poured into a mould and pressed to a flat board at a load of nine tons for 60 min at room temperature to produce composite board samples. The size of the sample fabricated board was 150 mm × 150 mm × 6 mm. The rectangular board was cut to test samples and stored in a zip lock bag.

### 2.4. Characterization

#### 2.4.1. Microstructure Properties

The crystallite size of the coconut shell particle (powder) was evaluated by using X-ray diffraction (XRD), Shimadzu type D6000 (manufactured by Shimadzu Company, Tokyo, Japan). Its crystallite size (D) was calculated by using Equation (1).

(1) $$D=\frac{k.\lambda }{\beta .Cos(\theta )}$$

where *λ* is 0.15406 nm (the wavelength of X-ray), *k* is a constant that is 0.95, *θ* is the Bragg angle of XRD peak in degrees, *β* is the full width at half maximum in radian.

Transmission Electron Microscopy (TEM), type FEI-Tecnai G2-20-S-Twin (manufactured by FEI Company, Hillsboro, OR, USA) was also used to analyze the coconut shell particle. The coconut shell nanoparticles were dispersed in water and sonicated. A droplet was taken and placed on a copper foil grid. The droplet on the copper grid was stained with uranyl acetate and placed in a copper grid to observe the coconut shell nanoparticle.

The morphological properties of the flexural fractured surfaces of the coconut shell biocomposite samples were analyzed with Scanning Electron Microscope—Energy Dispersive Spectroscopy (SEM-EDS) type JSM 6510LA (manufactured by JOEL Ltd., Tokyo, Japan). The fractured surface of the biocomposite was observed under light emission of the SEM after carbon-coated to get the fractured surface images. Furthermore, the elemental composition by volume was measured with EDS. Atomic Force Microscopy (AFM) type easyScan 2 (manufactured by Nanosurf, Liestal, Switzerland) was used to observe the morphological topography features of the coconut shell biocomposite and to observe the miscibility.

#### 2.4.2. Mechanical Properties

The samples were prepared by using ASTM D790 for the test of the mechanical property of the biocomposite. The Universal Testing Machine type HT-2402 (manufactured by Hung Ta Company, Taichung City, Taiwan) was used to measure the mechanical property of the samples. The flexural strength of the sample was determined by using Equation (2).

(2) $$FS=\frac{3.P.S}{2.b.t^{2}}$$

where *t* is the thickness of the sample, *b* is the width of the sample, *S* is the span, and *P* is the maximum load, and *F.S.* is flexural strength. The flexural modulus of the sample is determined by using Equation (3).

(3) $$FM=(\frac{\Delta F}{\Delta y})\frac{S^{3}}{4.b.t^{3}}$$

where (Δ*F*/Δ*y*) is the slope of the force against deformation, *t*, *b*, and *S* are the same as in Equation (1), F.M. is the flexural modulus of the sample.

#### 2.4.3. Physical Properties

The density (*ρ*) of the coconut shell nanoparticle composite was determined by using Equation (4).

(4) $$\rho =\frac{m}{V}$$

where *m* is the mass of the sample, *V* is the volume of the sample. The thickness swelling (*T.S.*) of the sample is calculated by using Equation (5).

(5) $$TS=\frac{T_{w}-T_{d}}{T_{d}}\times 100\%$$

where *T_d_* is the sample thickness in dry condition (before immersion in water), and *Tw* is the sample thickness after 24 h immersion in water.

Porosity is one of the physical properties to determine the number of pores in a particular material, which is the ratio between the pore volumes with the overall volume of that material. By assuming the pore volume is equal to the volume of water absorbed, the porosity (*P*) of the composite sample can be determined by using Equation (6).

(6) $$P=\left(\frac{M_{sat}-M_{dry}}{V_{bulk}}\right)\times \frac{1}{\rho_{water}}\times 100\%$$

Where *M_dry_* is the mass of the sample in dry condition, *M_sat_* is the sample mass after immersing the sample in water for 24 h, *V_bulk_* is the overall volume of the sample, *ρ_water_* is the density of water.

## 3. Results and Discussion

### 3.1. Microstructure Properties

**Figure 1:**
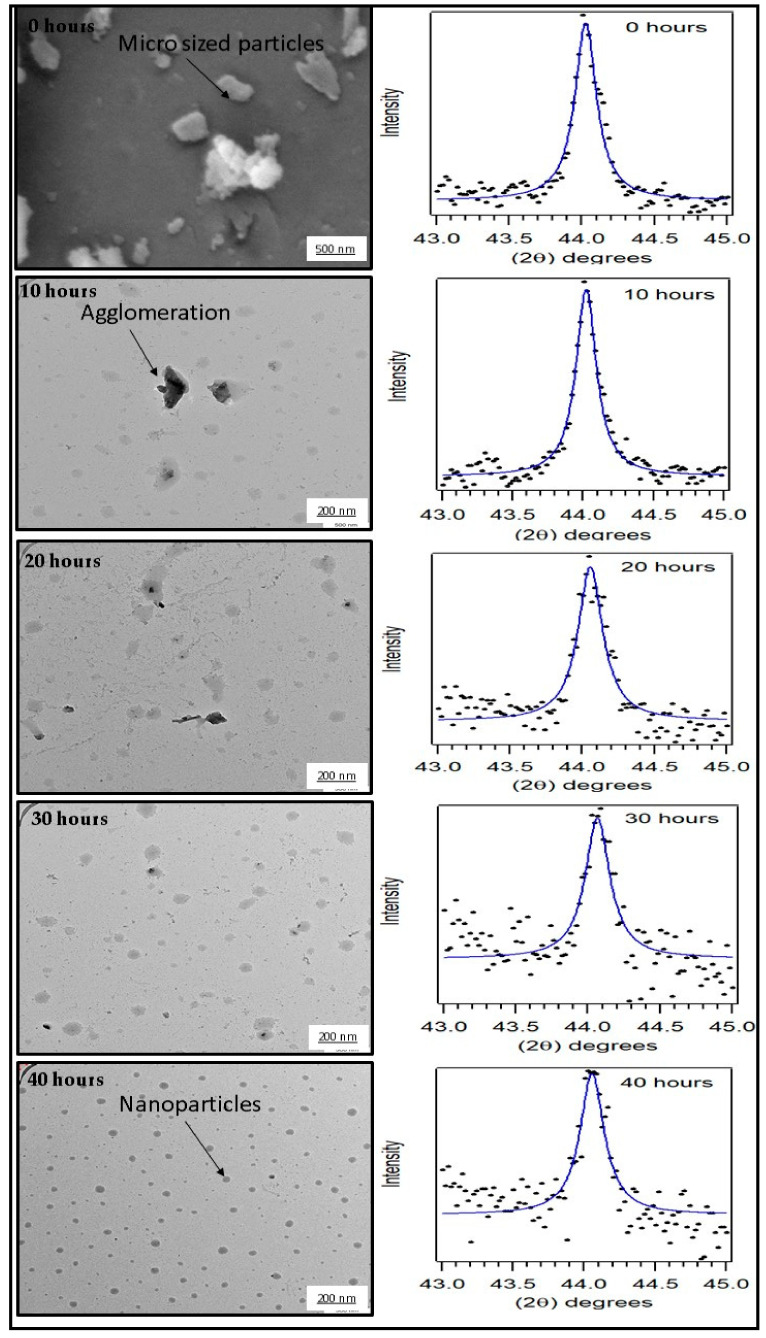
TEM and XRD data (Fe_2_O_3_ peak) of coconut shell particle (dots) at 0, 10, 20, 30, and 40 h.

Figure 1 showed the result of the TEM and X-ray diffraction analysis of the coconut shell particles at different milling times. The morphology of coconut shell particles was observed with Transmission Electron Microscopy (TEM). The TEM image for 0 h milling time (before milling) showed the microparticles of coconut shells as round shaped particles (spherical shape). The images of the TEM for coconut shell particles after milling for 10, 20, 30, and 40 h are seen as tiny spherical nanoparticles. It was observed from the images that the number of small particles increases after milling. Furthermore, some agglomerations of particles were observed with TEM of 0, 10, and 20 h. As the particle milling time increases, the cluster of particles disappeared as no agglomeration was observed with 30 and 40 h. These clusters were also observed in previous studies [29,30,31]. The TEM images confirmed the production of nanoparticles from the milling operation.

The X-ray diffraction (XRD) data of coconut shell particles for various milling times have also been reported in the previous work where the Fe_2_O_3_ peak has been identified at the Bragg angles of 44.05 degrees [27]. However, the crystallite size of the coconut shell particle was still unknown. The previous work found that the duration of milling significantly influenced the composition of Fe_2_O_3_in the coconut shell particles. In this present work, we have used the XRD data (Fe_2_O_3_ peak) for various milling times to examine whether the size of the crystallite of coconut shell particle is influenced or not by the duration of milling. Ball milling operation has been reported to have a significant influence on the crystallinity of materials [32]. Previous studies reported a similar trend in the crystallinity of the coconut shell, as reported in this study [33]. The Fe_2_O_3_ peak (XRD data) was fitted by using a Lorentzian function, as shown in Figure 1.

**Figure 2:**
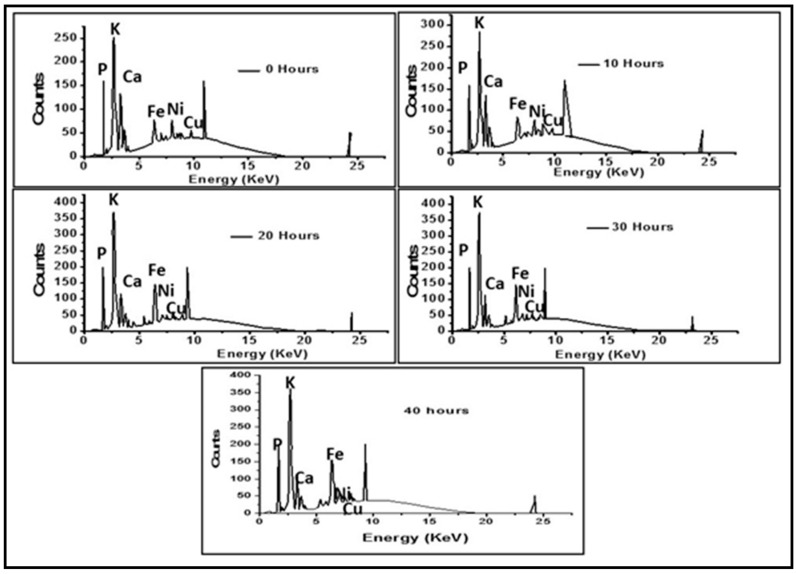
XRF analysis of the elemental composition of coconut shell particle at 0 h, 10 h, 20 h, 30 h, and 40 h.

The chemical composition change with the milling time of the coconut shell nanoparticle was confirmed with X-ray fluorescence analysis. The X-ray fluorescence (XRF) analysis result of the elemental composition of the coconut shell particles at 0, 10, 20, 30, 40 h milling time is shown in Figure 2. The composition of Fe_2_O_3_ was 13.6% before milling (200 mesh). After milling coconut shell particles for 10, 20, and 40 h, the Fe_2_O_3_ composition in the coconut shell particles increased. A similar observation was reported in previous work on the production of nanoparticle from coconut shells [27]. Generally, the analysis showed a change in elemental composition with an increase in milling time, as presented in Table 1. The elemental composition percentages of the major element shown by the peak of the graph changes with milling time. Potassium (K), calcium (Ca) decreased while the iron (Fe) increased. However, the phosphorus (P), nickel (Ni), and copper (Cu) show no consistent trend. The change in the chemical composition of the sample (Mo_2_NiB_2_-Ni) due to ball milling time has been observed previously by Zhang et al. [32]. They found that the composition of Mo changed from 61.26% (0 h milling time) to 53.97% (15 h milling time) [32]. The chemical composition changes due to the heat generated by grinding balls. The mechanical process can change the composition of a material if the process generates heat. The friction between the ball and the mill chamber during milling generated heat. The heat affected the coconut shell powder, and some of its composition changed [34]. The effect of the mechanical process can affect the composition of coconut shell because it is a natural filler and can be degraded by heat. Heat is a significant factor that affects the properties of natural fillers. Coconut shell is known to heat up because of its calorific value resulted in the composition variation [35]. The observation of changes in chemical composition may also be due to the area of contact between the particles. As the particle surface area increases, it’s easier to detect the presence of the element present in the coconut shell.

**Figure 3:**
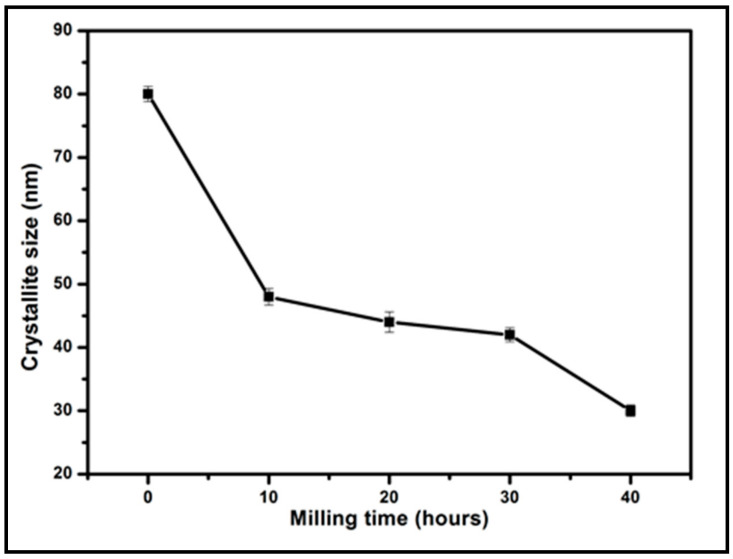
Crystallite size of coconut shell particles (with nano before milling at 0).

The fit of the XRD peak showed the full width at half maximum (β) for each milling time. The crystallite size of the coconut shell particle for each milling time was obtained with Equation (1). For 0 h of milling time, the crystallite size is found to be 49.5 nm. For 10 and 20 h milling time, the crystallite size of coconut shell particles decreases to 48.1 nm and 43.2 nm, respectively. The crystallite size keeps decreasing to 41.1 nm for 40 h duration of milling. Figure 3 displays the trend of the crystallite size of coconut shell particles with respect to the duration of milling of coconut shell particles. The duration of milling affected the crystallite size of the coconut shell particle significantly. As the duration of milling increases, the crystallite size decreases substantially. This trend was also observed in the previous studies of coconut shell particles by Bello et al. [36].

**Figure 4:**
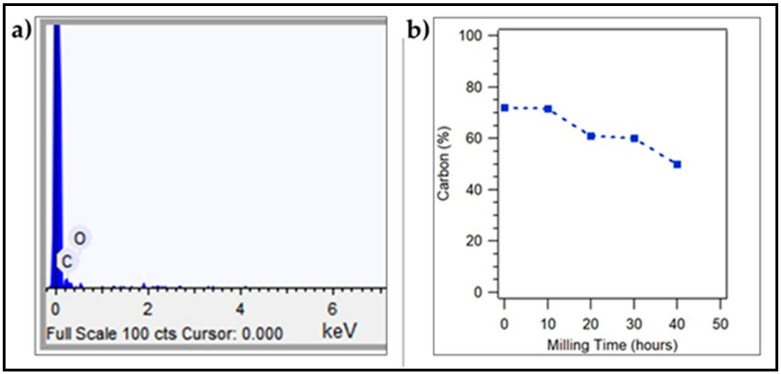
(**a**) SEM-EDS spectrum of coconut shell particles, (**b**) percentage of carbon in coconut shell particles.

The composition of coconut shell particles has also been analyzed using SEM-EDS. The EDS spectrum of the coconut shell particle is shown in Figure 4a. The dominant elements in the coconut shell particles are carbon and oxygen. Before milling the coconut shell particle (0 h of milling time), there was 72.0% of carbon elements observed in the coconut shell particle. For 10 h of milling time, there was 71.6% of carbon observed. As the milling time increases to 20 h, the percentage of carbon reduces to 61.0%. For 30 h and 40 h of milling time, the percentage of carbon becomes 60.1% and 50.0% in the coconut shell particles, respectively. This finding is very interesting as the carbon composition changes with the milling time of the samples. The percentage of carbon decreases as the milling time increases, as shown in Figure 4b. This result is similar to the report in a previous study, where the composition of carbon in the coconut shell was 50% [30].

**Figure 5:**
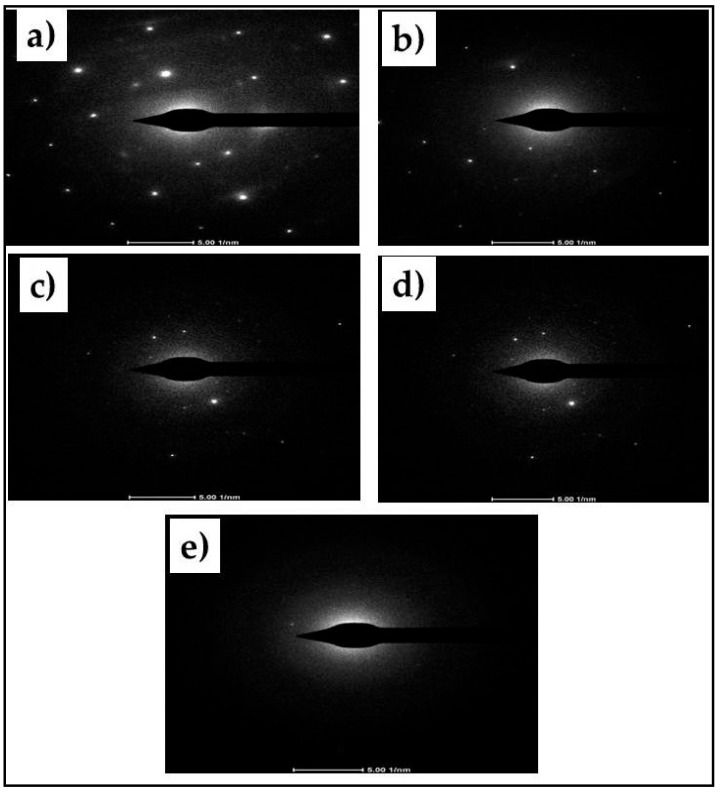
The S.A.E.D. images of coconut shell particles for various milling times, (**a**) for 0 h, (**b**) for 10 h, (**c**) for 20 h, (**d**) for 3 h, and (**e**) for 40 h.

Selected area electron diffraction (S.A.E.D.) of coconut shell particles was also performed. The results are shown in Figure 5. The result of 0 h milling is shown in Figure 5a where the resulting image shows order bright spots, indicating that the coconut shell particles are crystallite.

As the sample was milled, the brightness of spots became less, as shown in Figure 5b–e because of reducing the crystallite size of the coconut shell particle. These S.A.E.D. results are consistent with the XRD results, where increasing the milling will decrease the crystallite size.

The composite flexural fracture surfaces were analyzed by scanning electron microscope (SEM), and the results are shown in Table 2. The composite has some porosities and agglomerations majorly due to the microsize of the coconut shell particles for 0 h milling time. A similar situation was observed for 10 h of milling time, but the porosity is reduced. Some coconut shell particles were detached from the epoxy matrix. However, at 30 and 40 h of milling time, the composite had fewer porosities and coconut shell particles were mixed well with the matrix. The porosity and agglomeration were reduced with milling time due to the reduction of the particle sizes from micro to nanometre. The uniform mix and compatibility of the blend resulted in improved properties. Furthermore, a reduction in particle sizes improved the contact area between the coconut shell and the epoxy resin, resulting in good miscibility of the blend.

The atomic force microscopy (AFM) has also been used to analyze the morphology of the composites. Similar to SEM, the composite samples were cut to analyze the side surfaces. Table 2 shows the AFM image of the coconut shell composite for various milling times. The AFM image shows that the composite surface of after milling the particles has less corrugation and a more flat surface compared to the composite surface for 0 h of milling time. This is seen in the increased smoothness of the surface topography with increased milling time. These results are in agreement with the SEM results as the AFM images showed improved miscibility between the coconut shell particles and the epoxy with the milling time. The smooth topography is a result of the increase in surface area of the nanoparticles of the coconut shell and, in turn, resulted in uniform miscibility of the composite.

However, at lower milling time, the surface topography is rough, which was also noticed with the result of the fractured surface images of the SEM. This finding implies that the bonding between matrix and filler improves as the milling time of coconut shell particle increases. This result suggests that the mechanical and physical properties of the composite will be improved as the milling time of coconut shell particles is increased.

### 3.2. Mechanical Properties

**Figure 6:**
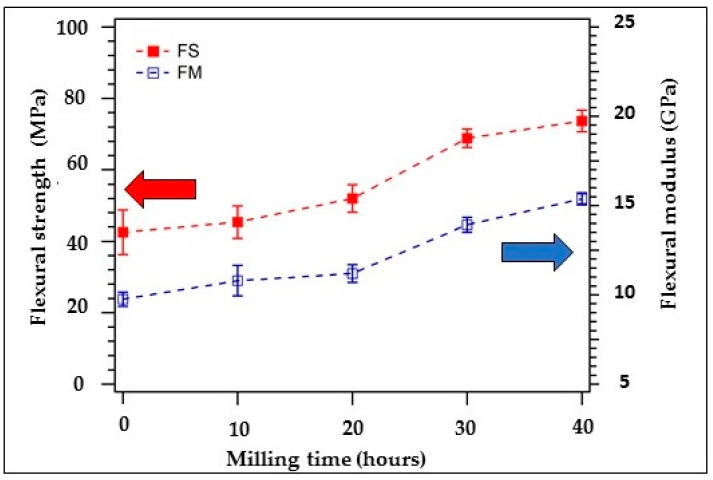
Flexural strength and modulus of the biocomposite for various milling times.

The result of the flexural strength and flexural modulus of the composite is plotted in Figure 6. For 0 h milling time of coconut shell particle (200 mesh of coconut shell particle size), the flexural strength is found to be 43.28 MPa. When the coconut shell particle is milled for 10 h, flexural strength increases slightly to 44.67 MPa. The flexural strength continues to increase as the milling time is increased. For milling time 20 h, 30 h, and 40 h, the flexural strength becomes 58.98 MPa, 64.41 MPa, and 68.83 MPa, respectively. The flexural strength of the composite significantly depends on the milling times of coconut shell particles, which implies that the size of the coconut shell particle influences the flexural strength of the composite. As the amount of milling time of coconut shell particle increases, the flexural strength of the composite increases. The flexural modulus of the biocomposite is shown in Figure 6 (unfilled squares). For 0 h milling time, the flexural modulus of the biocomposite is found to be 9.90 GPa. As the coconut shell particles are milled for 10 h, the flexural modulus increases to 11.32 GPa. The flexural modulus increased to 12.57 Gpa, 14.50 GPa and 15.50 GPa when the coconut shell particles were milled for 20 h, 30 h, and 40 h, respectively.

**Figure 7:**
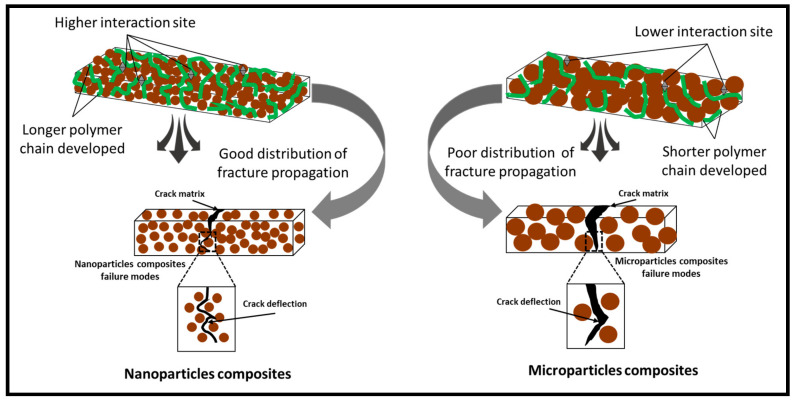
Schematic diagram of crystallite effect on the mechanical properties of the coconut shell nanoparticle composite board.

The flexural modulus of the composite is also strongly dependent on the milling time of coconut shell particles. This behavior is related to the crystallite size of coconut shell particles. As the milling time is increased, the crystallite size decreases. The surface contact between the matrix and filler (particles) increases. Then, the bonding between matrix and filler improves as shown by SEM and AFM in the previous section. As a result, the mechanical properties of the composite improve. Figure 7 showed the pictural explanation of the internal structure of the composite. Larger particles resulted in poor miscibility of the composite, while smaller crystallite improved the miscibility. This mechanism drawn in Figure 7 confirmed the result obtained from the SEM and AFM images.

The highest value of the flexural strength of the composite nano board from this study is 68.83 MPa, where the composition of the composite was 85 vol.% of coconut shell particle and 15 vol.% of epoxy. Somashekhar et al. studied the coconut shell composite, but the composition was 30 wt.% of coconut shell particle and 70 wt.% of epoxy [16]. Their report showed that the flexural strength of their composite was 40 Mpa, which is lower than the result. Durowaye prepared the coconut shell composite by using a polyester matrix, and the composition was 30 wt.% of coconut shell particle and 70 wt.% of polyester [17]. The report of their study showed that the flexural strength of their composite was only 15.78 Mpa, which is smaller than the result. The flexural strength of the composite from this study is also above the flexural strength of rice straw composite using epoxy resin matrix (its flexural strength was 32.46 MPa) [37], where the composition of the composite was 95 vol.% of rice straw particle and 5 vol.% of epoxy. The highest value of flexural modulus of the biocomposite is 15.50 Gpa, which is above the value in previous studies (39 wt.% of coconut shell composite and 61 wt.% of epoxy) [4,18,37]. This is probably due to the size of the nanoparticles of the coconut shell used in this study compared with micro-sized used in previous studies.

According to ANSI Standard (ANSI 208.1-2009), the flexural modulus and flexural strength requirement forthe highest grade of particleboard is 21.1 MPa and 2.475 GPa, respectively [38]. The coconut shell composite in this study has flexural strength in the range of 43.28 to 68.83 Mpa and the flexural modulus of the composite is found in the range of 9.9 to 15.50 GPa. Thus, it showed that the composites (i.e., composites for coconut shell particles milled for 30 and 40 h) fulfil the ANSI requirements for H-3 grade (the highest grade of particleboard).

### 3.3. Physical Properties

**Figure 8:**
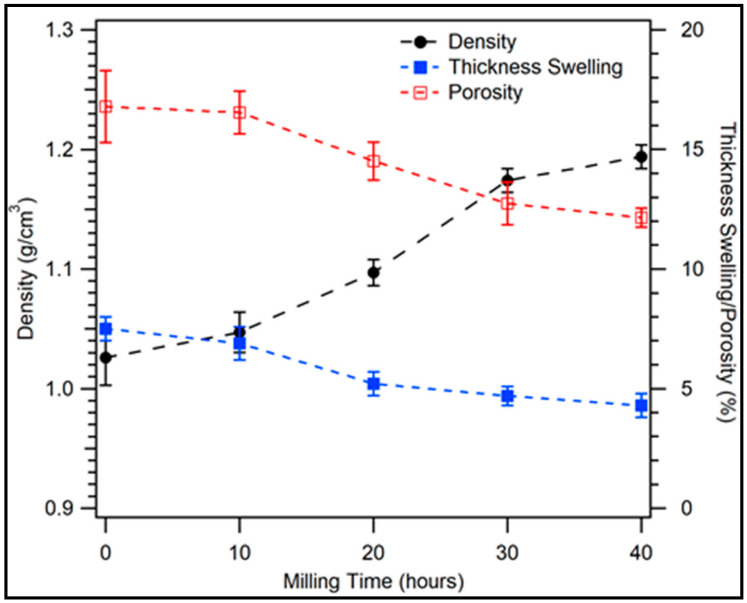
Density, thickness swelling, and porosity of the biocomposite for various milling times.

The density, thickness swelling, and porosity of the composite for various milling times of coconut shell particles were measured. The results are shown in Figure 8. For 0 h milling time, the density of coconut shell composite is 1.030 g/cm^3^. The density increases to 1.047 g/cm^3^ as the coconut shell particle is milled for 10 h. The density of coconut shell composite increases to 1.097 g/cm^3^ as the particle is milled for 20 h, and keeps increasing to 1.194 g/cm^3^ for 40 h milling time. The density of coconut shell composite increases as the milling time is increased. This condition is related to the reduction of particle size as the duration of milling is increased.

The density of the composite from this study is about the same as the density of coconut shell composites from previous studies by Singh et al. [15] and Bhaskar et al. [39]. Comparing to the rice straw composite [37], the density of the coconut shell composite is much higher than the density of the rice straw composite. This happens because the density of coconut shell particles is greater than the density of rice straw particles.

The thickness swelling of the composite for the various durations of milling is shown in Figure 8. It was observed that the thickness swelling of the composite for 0 h milling time of coconut shell particle is 7.5%. As the coconut shell particle is milled for 10 h, the thickness swelling of the composite slightly reduces to 6.9%. For 20 and 30 h of milling times, the thickness swelling reduces to 5.2% and 4.7%, respectively. The thickness swelling decreases to 4.3% as the milling time is increased to 40 h. In general, the swelling thickness of the composite decreases as the duration of milling of coconut shell particles is reduced. This happens because the density of the composite increases as the milling time is increased. Bamboo epoxy composite has 4% thickness swelling [40], which is about the same as that of the coconut shell epoxy composite from this study for 40 h duration of milling.

The porosity of the composite for various milling times is shown in Figure 8 (unfilled squares). For 0 h of milling time, the porosity is 16.8%. The percentage of porosity of the composite decreases to 14.5% for 20 h duration of milling. The porosity of the composite decreases to 12.2% for 40 h milling duration of coconut shell particles. In general, the porosity decreases when the duration of milling is increased, which is in agreement with the thickness swelling. This behavior is related to the density of the composite. When the density of the composite increases, the number of the void (empty space) in the composite decreases. As a result, the porosity and thickness swelling of the composite decrease.

## 4. Conclusions

In this study, the coconut shell nanoparticle composite using the epoxy resin matrix has been prepared and characterized. The crystallite size of coconut shell particles was found to be reduced as the duration of milling was increased. As observed by TEM, the size of coconut shell particles was reduced when the duration of milling was increased. The properties of the composite improved as the duration of milling was increased. The improvement of mechanical performance was related to the increase of bonding between epoxy resin (matrix), and coconut shell nanoparticles, as observed by SEM and AFM. The physical properties of the composite also improved as the duration of milling was increased. The composite fulfils the ANSI requirements for the H-3 grade composite board. Thus, the coconut shell nanoparticle composite board can be fabricated commercially for particleboard, furniture, and other applications. The finding revealed that the properties (microstructure, mechanical, and physical) of coconut shell nanoparticle composite were affected significantly by milling time. The finding from this study suggested that the size of filler plays an important role in improving the composite properties.

## Figures and Tables

**Table 1:** Elemental composition variation of coconut shell particle with milling time.

Miling Time	K (wt.%)	Ca (wt.%)	Fe (wt.%)	P (wt.%)	Cu (wt.%)	Ni (wt.%)
0 h	48.6	25.4	12.2	5.3	3.1	0.8
10 h	42.1	21.1	28.5	4.1	3.3	1.2
20 h	38.8	19.2	28.6	4.4	3.7	2.1
30 h	38.7	18.5	30.0	3.9	3.6	2.0
40 h	38.0	17.4	32.2	4.2	3.6	1.9

**Table 2:** SEM and AFM images of the coconut shell nanoparticle at milling time 0, 10, 20, 30, 40 h.

Milling Times	SEM	AFM
**0 h**	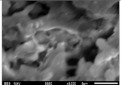	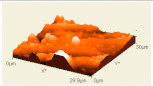
**10 h**	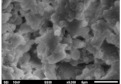	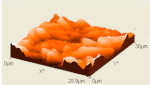
**20 h**	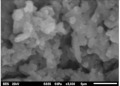	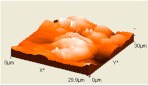
**30 h**	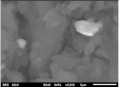	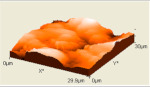
**40 h**